# Clinical burden of invasive *Escherichia coli* disease among older adult patients treated in hospitals in the United States

**DOI:** 10.1186/s12879-023-08479-3

**Published:** 2023-08-22

**Authors:** Luis Hernandez-Pastor, Jeroen Geurtsen, Bryan Baugh, Antoine C. El Khoury, Nnanya Kalu, Marjolaine Gauthier-Loiselle, Rebecca Bungay, Martin Cloutier, Michal Sarnecki, Elie Saade

**Affiliations:** 1 Global Market Access, Vaccines Janssen Pharmaceutica NV, Turnhoutseweg 30, Beerse, B-2340 Belgium; 2 Molecular Bacteriology & Bacterial Epidemiology, Janssen Vaccines & Prevention BV, Archimedesweg 4, Leiden, 2333 CN The Netherlands; 3 Global Medical Affairs, Janssen Research & Development, LLC, 1000 U.S. Route 202 South, Raritan, NJ 08869 USA; 4 Global Market Access, Janssen Global Services, LLC, 1000 U.S. Route 202 South, Vaccines, Raritan, NJ 08869 USA; 5 US Vaccines Medical Affairs, Janssen Scientific Affairs, LLC, 1125 Trenton-Harbourton Road, 08560 Titusville, NJ USA; 6 Health Economics and Outcomes Research, Analysis Group, Inc, 1190 avenue des Canadiens- de-Montréal, Tour Deloitte, Suite 1500, H3B 0G7 Montreal, QC Canada; 7 Clinical Development, Janssen Vaccines, Rehhagstrasse 79, 3018 Bern, Switzerland; 8 Department of Medicine, Case Western Reserve University, Health Education Campus, 9501 Euclid Ave, 44106 Cleveland, OH USA

**Keywords:** Invasive *Escherichia coli* disease, Medical resource utilization, Treatment patterns, Antibiotic resistance, Case fatality rate

## Abstract

**Background:**

Invasive extraintestinal pathogenic *Escherichia coli* disease (IED) can lead to severe outcomes, particularly among older adults. However, the clinical burden of IED in the U.S. has not been well characterized.

**Methods:**

IED encounters among patients ≥ 60 years old were identified using the PINC AI™ Healthcare Database (10/01/2015–03/31/2020) by either a positive *E. coli* culture in blood or another normally sterile body site and ≥ 1 sign of systemic inflammatory response syndrome or signs of sepsis, or a positive *E. coli* culture in urine with urinary tract infection and signs of sepsis. Medical resource utilization, clinical outcomes, and *E. coli* isolate characteristics were descriptively reported during the first IED encounter and during the following year (observation period).

**Results:**

Overall, 19,773 patients with IED were included (mean age: 76.8 years; 67.4% female; 78.5% with signs of sepsis). Most encounters involved community-onset IED (94.3%) and required hospitalization (96.5%; mean duration: 6.9 days), with 32.4% of patients being admitted to the intensive care unit (mean duration: 3.7 days). Most *E. coli* isolates were resistant to ≥ 1 antibiotic category (61.7%) and 34.4% were resistant to ≥ 3 antibiotic categories. Following their first IED encounter, 34.8% of patients were transferred to a skilled nursing/intermediate care facility, whereas 6.8% had died. During the observation period, 36.8% of patients were rehospitalized, 2.4% had IED recurrence, and in-hospital death increased to 10.9%.

**Conclusions:**

IED is associated with substantial clinical burden at first encounter with considerable long-term consequences. Findings demonstrate the need for increased IED awareness and highlight potential benefits of prevention.

**Supplementary Information:**

The online version contains supplementary material available at 10.1186/s12879-023-08479-3.

## Background

*Escherichia coli* (*E. coli*) are a large and diverse group of bacteria that can be found as part of the normal human intestinal flora. Pathogenic *E. coli*, both intestinal pathogenic *E. coli* (InPEC) as well as extraintestinal pathogenic *E. coli* (ExPEC), comprise *E. coli* strains that may cause infections with potentially severe complications, including death [1]. Indeed, *E. coli* is a leading cause of community-acquired sepsis, a life-threatening condition that is among the main reasons for hospitalization and death in the U.S. [2–6], particularly among older patients [7].

Pathogenic *E. coli* can emerge to infect normally sterile body sites and lead to invasive *E. coli* disease (IED), also known as invasive ExPEC disease, which comprises sepsis (including sepsis due to urinary tract infection [UTI], i.e., urosepsis), bacteremia, peritonitis, meningitis, and other infectious syndromes [2, 8–10]. A recent meta-analysis reported that the incidence rate for *E. coli* bacteremia rises progressively beyond 60 years of age, from 110 to 100,000 personyears among adults 60–69-year-old to 319 per 100,000 person-years among those 80 years or older [11]. Older patients with *E. coli* bacteremia are also more likely to have antibiotic-resistant isolates than those aged 18–64 years [12]. These findings are of particular importance given that *E. coli* is the most common pathogen linked to deaths associated with antibiotic resistance [13]. Taken together, these studies suggest that older adults may be at greater risk of developing IED and may be more challenging to manage due to the increased likelihood of antibiotic resistance.

Despite its clinical importance, the burden of IED in the U.S., particularly among older adults, is not well characterized. Furthermore, while the epidemiology of IED and patterns of antibiotic resistance have been previously described, including in the U.S. [14], various definitions of IED are used across studies. Therefore, the aim of this study was to describe and characterize the short-term as well as the longer-term outcomes following IED among patients 60 years and older hospitalized in the U.S. using an inclusive definition of IED, which encompassed cases beyond *E. coli* bacteremia.

## Methods

### Data source

This study used data from the PINC AI™ Healthcare Database (PHD). The data period spanned from October 1, 2015 – March 31, 2020 to include recent data, while focusing on presumed pre-COVID period to reduce risk for over-estimation of the burden due to additional in-hospital health-care services that may have been provided as a result of COVID infections in older patients. The PHD comprises detailed inpatient services from patients admitted to a representative set of > 1,000 U.S. hospitals nationwide and includes admission-level information (e.g., patient characteristics, primary and secondary admitting diagnoses), detailed day-of-service billing information during hospitalizations (e.g., inpatient procedures and medications used by day of stay), and discharge-level data (e.g., length of stay, discharge status) [15]. Data are de-identified and comply with the requirements of the Health Insurance Portability and Accountability Act of 1996; therefore, no review by an institutional review board was required.

### IED case identification and subtype

**Fig. 1:**
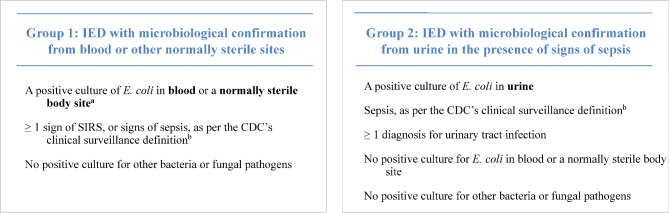
IED type **Abbreviations**: CDC: Centers for Disease Control; IED: invasive *Escherichia coli* disease **Notes:Notes**: ^a^ Normally sterile body sites include cerebrospinal fluid, pleural fluid (chest fluid, thoracentesis fluid), peritoneal fluid (abdominal fluid, ascites), pericardial fluid, bone (including bone marrow), joint fluid (synovial fluid, fluid, needle aspirate, or culture of any specific joint such as knee, ankle, elbow, hip, wrist), and internal body sites (lymph node, brain, heart, liver, spleen, vitreous fluid, kidney, pancreas, ovary, vascular tissue, deep wound) ^b^ The sepsis clinical surveillance definition utilizes an algorithm defined by Rhee et al. (2017) and details and diagnosis codes were updated using the CDC’s *Hospital Toolkit for Adult Sepsis Surveillance (March 2018).* The algorithm was validated using medical records from 510 randomly selected hospitalizations, stratified into those that did and did not meet sepsis surveillance criteria

IED encounters were classified as either Group 1 IED, corresponding to IED with a positive *E. coli* culture in blood or other normally sterile body sites and ≥ 1 sign of systemic inflammatory response syndrome (SIRS) or signs of sepsis (as per the Centers for Disease Control [CDC] clinical surveillance definition [16]) without positive culture for other bacterial or fungal pathogens, or Group 2 IED, corresponding to IED with microbiological confirmation from urine in the presence of signs of sepsis (as per the CDC clinical surveillance definition [16]) and a diagnosis code for UTI without a positive culture for other bacterial or fungal pathogens (Fig. 1). IED encounters that met the definition for both Group 1 and Group 2 IED were classified in Group 1. In addition, among patients classified in Group 1, the subgroup of patients that had signs of sepsis was identified (i.e., Group 1 IED with sepsis).

### Study design and sample selection

A retrospective study design was used (Supplementary Figure S1) whereby the *index date* for a given patient was the date of the first positive *E. coli* culture during the first documented IED encounter (i.e., *index encounter)*, and the *observation period* was defined as the 12-month period following the index date. Patients were included in the study if they had ≥ 1 IED encounter and were ≥ 60 years of age as of the index date. To increase the likelihood of capturing the first IED encounter as of the index date and to ensure an adequate observation period, the IED encounter was required to occur in a hospital that contributed microbiology data to the database continuously for ≥ 6 months before and ≥ 12 months after the index date.

### Measures, outcomes, and statistical analyses

Patient and hospital characteristics were descriptively reported, as well as the characteristics and course of the index encounter which included the point of origin (e.g., clinic, transfer from another hospital), the IED onset (hospital or community, defined respectively based on the date of the positive *E. coli* culture ≥ 3 days vs. ≤2 days after hospital admission, and whether community-onset IED was healthcare-associated [17, 18]), the type of encounter (inpatient stay, emergency room visit, or outpatient hospital visit), the type of IED (i.e., Group 1, Group 1 with sepsis, or Group 2), infection type (e.g., urosepsis with/without bacteremia, meningitis; Supplementary Table S1), IED-related treatments, and discharge status. Patterns of antibiotic resistance, including multi-drug resistance (MDR), were explored among IED encounters for which antibiotic susceptibility tests were available. MDR was defined as isolates resistant to ≥ 1 agent in ≥ 3 relevant antibiotic categories (Supplementary Table S2), based on a joint initiative by the European and U.S. CDC [19]. Trends in antibiotic resistance over time between 2015 and 2019 were also assessed. IED recurrences, defined as an encounter for IED with a gap of ≥ 14 days from the last positive *E. coli* culture from a prior IED, were assessed during the 12month observation period. Analyses were conducted overall and stratified by type of IED (i.e., Group 1, Group 1 with sepsis, Group 2). Stratified analyses were also conducted by patient age (i.e., 60–75 or ≥ 75 years old), onset of IED (i.e., hospital-onset, community-onset), and MDR status. Statistical comparisons for these variables were conducted using Wilcoxon rank-sum and Chi-square tests. All analyses were performed using SAS Enterprise software programs (version 7.1).

## Results

### Study sample and characteristics

A total of 19,773 patients with ≥ 1 IED encounter in a U.S. hospital were included in the study Supplementary Figure S2). The characteristics of patients with IED are reported in Table 1. In the overall sample, mean age was 76.8 years, 67.4% were female, and 82.1% were White. The most common comorbidities at the index date were high blood pressure (80.2%), renal disease (33.0%), and congestive heart failure (29.3%).

**Table 1:** Patient and hospital characteristics on the index date

	All patients	Type of IED at index date					
		Group 1	Group 2	P-value	Group 1 with sepsis	Group 2	P-value
	N = 19,773	N = 10,235	N = 9,538		N = 5,978	N = 9,538	
**Demographic characteristics**							
**Age, mean ± SD [median]**	**76.8 ± 8.9 [77.0]**	75.9 ± 8.9 [76.0]	77.8 ± 8.8 [79.0]	< 0.0001	76.2 ± 8.9 [76.0]	77.8 ± 8.8 [79.0]	< 0.0001
< 65 years old, n (%)	2,231 (11.3)	1,336 (13.1)	895 (9.4)	< 0.0001	735 (12.3)	895 (9.4)	< 0.0001
65–74 years old, n (%)	5,876 (29.7)	3,326 (32.5)	2,550 (26.7)	< 0.0001	1,898 (31.7)	2,550 (26.7)	< 0.0001
75–84 years old, n (%)	6,510 (32.9)	3,256 (31.8)	3,254 (34.1)	0.0006	1,936 (32.4)	3,254 (34.1)	0.0262
≥ 85 years old, n (%)	5,156 (26.1)	2,317 (22.6)	2,839 (29.8)	< 0.0001	1,409 (23.6)	2,839 (29.8)	< 0.0001
**Gender, n (%)**							
Female	13,321 (67.4)	6,016 (58.8)	7,305 (76.6)	< 0.0001	3,390 (56.7)	7,305 (76.6)	< 0.0001
Male	6,451 (32.6)	4,219 (41.2)	2,232 (23.4)	< 0.0001	2,588 (43.3)	2,232 (23.4)	< 0.0001
Unknown	1 (0.0)	0 (0.0)	1 (0.0)	-	0 (0.0)	1 (0.0)	-
**Race, n (%)**							
White	16,234 (82.1)	8,302 (81.1)	7,932 (83.2)	0.0002	4,768 (79.8)	7,932 (83.2)	< 0.0001
Black	1,799 (9.1)	831 (8.1)	968 (10.1)	< 0.0001	535 (8.9)	968 (10.1)	0.0140
Asian	646 (3.3)	434 (4.2)	212 (2.2)	< 0.0001	284 (4.8)	212 (2.2)	< 0.0001
Other	909 (4.6)	569 (5.6)	340 (3.6)	< 0.0001	339 (5.7)	340 (3.6)	< 0.0001
Unknown	185 (0.9)	99 (1.0)	86 (0.9)	0.6320	52 (0.9)	86 (0.9)	0.8373
**Comorbidities**							
**CCI score**,^**a**^**mean ± SD [median]**	**2.5 ± 2.1 [2.0]**	2.2 ± 2.1 [2.0]	2.8 ± 2.1 [2.0]	< 0.0001	2.5 ± 2.2 [2.0]	2.8 ± 2.1 [2.0]	< 0.0001
≥ 3, N (%)	8,398 (42.5)	3,730 (36.4)	4,668 (48.9)	< 0.0001	2,555 (42.7)	4,668 (48.9)	< 0.0001
**CCI comorbidities** ^**b**^							
AIDS/HIV	21 (0.1)	9 (0.1)	12 (0.1)	0.4139	7 (0.1)	12 (0.1)	0.8799
Cancer	2,102 (10.6)	1,217 (11.9)	885 (9.3)	< 0.0001	765 (12.8)	885 (9.3)	< 0.0001
Any malignancy, including lymphoma and leukemia except malignant neoplasm of the skin	1,295 (6.5)	793 (7.7)	502 (5.3)	< 0.0001	492 (8.2)	502 (5.3)	< 0.0001
Metastatic solid tumor	807 (4.1)	424 (4.1)	383 (4.0)	0.6516	273 (4.6)	383 (4.0)	0.0968
Cerebrovascular disease	1,811 (9.2)	626 (6.1)	1,185 (12.4)	< 0.0001	426 (7.1)	1,185 (12.4)	< 0.0001
Chronic pulmonary disease	5,036 (25.5)	2,324 (22.7)	2,712 (28.4)	< 0.0001	1,421 (23.8)	2,712 (28.4)	< 0.0001
Congestive heart failure	5,803 (29.3)	2,565 (25.1)	3,238 (33.9)	< 0.0001	1,762 (29.5)	3,238 (33.9)	< 0.0001
Dementia	4,465 (22.6)	1,536 (15.0)	2,929 (30.7)	< 0.0001	1,014 (17.0)	2,929 (30.7)	< 0.0001
Diabetes without chronic complications	4,266 (21.6)	2,243 (21.9)	2,023 (21.2)	0.2284	1,345 (22.5)	2,023 (21.2)	0.0580
Diabetes with complications	3,992 (20.2)	1,782 (17.4)	2,210 (23.2)	< 0.0001	1,226 (20.5)	2,210 (23.2)	0.0001
Hemiplegia or paraplegia	427 (2.2)	131 (1.3)	296 (3.1)	< 0.0001	95 (1.6)	296 (3.1)	< 0.0001
Mild liver disease	890 (4.5)	531 (5.2)	359 (3.8)	< 0.0001	381 (6.4)	359 (3.8)	< 0.0001
Moderate or severe liver disease	430 (2.2)	242 (2.4)	188 (2.0)	0.0581	197 (3.3)	188 (2.0)	< 0.0001
Myocardial infarction	2,942 (14.9)	1,404 (13.7)	1,538 (16.1)	< 0.0001	979 (16.4)	1,538 (16.1)	0.6789
Peptic ulcer disease	360 (1.8)	139 (1.4)	221 (2.3)	< 0.0001	109 (1.8)	221 (2.3)	0.0380
Peripheral vascular disease	2,066 (10.4)	928 (9.1)	1,138 (11.9)	< 0.0001	604 (10.1)	1,138 (11.9)	0.0004
Renal disease	6,529 (33.0)	3,019 (29.5)	3,510 (36.8)	< 0.0001	2,034 (34.0)	3,510 (36.8)	0.0004
Rheumatic disease	933 (4.7)	482 (4.7)	451 (4.7)	0.9495	276 (4.6)	451 (4.7)	0.7490
**Other selected comorbidities** ^**b**^							
Cataracts	95 (0.5)	42 (0.4)	53 (0.6)	0.1398	23 (0.4)	53 (0.6)	0.1378
Glaucoma	625 (3.2)	277 (2.7)	348 (3.6)	0.0002	167 (2.8)	348 (3.6)	0.0038
Hearing problems	759 (3.8)	349 (3.4)	410 (4.3)	0.0012	207 (3.5)	410 (4.3)	0.0095
High blood pressure	15,849 (80.2)	7,974 (77.9)	7,875 (82.6)	< 0.0001	4,790 (80.1)	7,875 (82.6)	0.0001
Migraine/headache	392 (2.0)	224 (2.2)	168 (1.8)	0.0313	111 (1.9)	168 (1.8)	0.6633
Kidney stone	597 (3.0)	403 (3.9)	194 (2.0)	< 0.0001	259 (4.3)	194 (2.0)	< 0.0001
Benign prostate hyperplasia	2,086 (10.5)	1,294 (12.6)	792 (8.3)	< 0.0001	813 (13.6)	792 (8.3)	< 0.0001
**Hospital characteristics**							
**Number of beds, n (%)**							
≥ 500	6,032 (30.5)	2,972 (29.0)	3,060 (32.1)	< 0.0001	1,792 (30.0)	3,060 (32.1)	0.0059
**Region, n (%)**							
Midwest	4,721 (23.9)	2,386 (23.3)	2,335 (24.5)	0.0540	1,395 (23.3)	2,335 (24.5)	0.1042
Northeast	3,271 (16.5)	1,826 (17.8)	1,445 (15.1)	< 0.0001	1,089 (18.2)	1,445 (15.1)	< 0.0001
South	11,046 (55.9)	5,578 (54.5)	5,468 (57.3)	< 0.0001	3,198 (53.5)	5,468 (57.3)	< 0.0001
West	735 (3.7)	445 (4.3)	290 (3.0)	< 0.0001	296 (5.0)	290 (3.0)	< 0.0001
**Teaching hospital, n (%)**	7,909 (40.0)	3,998 (39.1)	3,911 (41.0)	0.0053	2,411 (40.3)	3,911 (41.0)	0.4062
**Population served, n (%)**							
Urban	16,638 (84.1)	8,494 (83.0)	8,144 (85.4)	< 0.0001	5,134 (85.9)	8,144 (85.4)	0.3913
Rural	3,135 (15.9)	1,741 (17.0)	1,394 (14.6)	< 0.0001	844 (14.1)	1,394 (14.6)	0.3913

**Abbreviations**: AIDS: acquired immunodeficiency syndrome; CCI: Charlson Comorbidity Index; HIV: human immunodeficiency virus; ICU: intensive care unit; IED: invasive *Escherichia coli* disease

**Notes:**^a^ Sources: Quan, H., Sundararajan, V., Halfon, P., et al. (2005). Coding algorithms for defining comorbidities in ICD-9-CM and ICD-10 administrative data. Medical Care, 43 [11], 1130–1139; Quan, H., Li, B., Couris, C. H., Fushimi, K., et al. (2011). Updating and validating the Charlson Comorbidity Index and score for risk. American Journal of Epidemiology, 173 [6], 676 − 672. Adjustment in Hospital Discharge Abstracts Using Data From 6 Countries. Medical care, 1130–1139

^b^ More than one option could apply (i.e., categories are not mutually exclusive)

### Characteristics of index encounters

Most index encounters were related to community-acquired IED (94.3%), and among those, 25.7% were healthcare-associated. The most frequent infection types were urosepsis without bacteremia (48.2%) and with bacteremia (29.3%; Table 2).

**Table 2:** Characteristics of the index encounter

	All patients	Type of IED at index date					
		Group 1	Group 2	P-value	Group 1 with sepsis	Group 2	P-value
	N = 19,773	N = 10,235	N = 9,538		N = 5,978	N = 9,538	
**Onset of IED, n (%)**							
Hospital-onset	1,125 (5.7)	402 (3.9)	723 (7.6)	< 0.0001	208 (3.5)	723 (7.6)	< 0.0001
Community-onset	18,648 (94.3)	9,833 (96.1)	8,815 (92.4)	< 0.0001	5,770 (96.5)	8,815 (92.4)	< 0.0001
Healthcare-associated community-acquired	4,787 (25.7)	2,289 (23.3)	2,498 (28.3)	< 0.0001	1,416 (24.5)	2,498 (28.3)	< 0.0001
Non-healthcare-associated community-acquired	13,861 (74.3)	7,544 (76.7)	6,317 (71.7)	< 0.0001	4,354 (75.5)	6,317 (71.7)	< 0.0001
**Infection type, n (%)**							
Urosepsis without bacteremia	9,538 (48.2)	0 (0.0)	9,538 (100.0)	-	0 (0.0)	9,538 (100.0)	-
Urosepsis with bacteremia	5,791 (29.3)	5,791 (56.6)	0 (0.0)	-	3,207 (53.6)	0 (0.0)	-
Cholangitis	299 (1.5)	297 (2.9)	2 (0.0)	< 0.0001	213 (3.6)	2 (0.0)	< 0.0001
Peritonitis	179 (0.9)	179 (1.7)	0 (0.0)	-	124 (2.1)	0 (0.0)	-
Other intra-abdominal infection	1 (0.0)	1 (0.0)	0 (0.0)	-	0 (0.0)	0 (0.0)	-
Neutropenic fever	166 (0.8)	127 (1.2)	39 (0.4)	< 0.0001	73 (1.2)	39 (0.4)	< 0.0001
Wound/surgical site infection	157 (0.8)	157 (1.5)	0 (0.0)	-	84 (1.4)	0 (0.0)	-
Osteomyelitis	39 (0.2)	39 (0.4)	0 (0.0)	-	27 (0.5)	0 (0.0)	-
Prostate biopsy-related infection	9 (0.0)	9 (0.1)	0 (0.0)	-	4 (0.1)	0 (0.0)	-
Meningitis	7 (0.0)	7 (0.1)	0 (0.0)	-	2 (0.0)	0 (0.0)	-
Complicated pneumonia	0 (0.0)	0 (0.0)	0 (0.0)	-	0 (0.0)	0 (0.0)	-
Other blood stream infections	3,774 (19.1)	3,774 (36.9)	0 (0.0)	-	2,334 (39.0)	0 (0.0)	-
Other	37 (0.2)	37 (0.4)	0 (0.0)	-	25 (0.4)	0 (0.0)	-
**Type of encounter, n (%)**							
Inpatient stay	19,084 (96.5)	9,546 (93.3)	9,538 (100.0)	< 0.0001	5,978 (100.0)	9,538 (100.0)	-
Duration of inpatient stay (days), mean ± SD [median]	6.9 ± 5.7 [5.0]	6.4 ± 5.6 [5.0]	7.4 ± 5.8 [6.0]	< 0.0001	7.2 ± 5.9 [6.0]	7.4 ± 5.8 [6.0]	0.3166
Duration of inpatient stay after the first positive *E. coli* culture, mean ± SD [median]	6.5 ± 5.1 [5.0]	6.1 ± 4.9 [5.0]	6.9 ± 5.2 [6.0]	< 0.0001	6.9 ± 5.3 [6.0]	6.9 ± 5.2 [6.0]	0.0587
Emergency room visit	554 (2.8)	554 (5.4)	0 (0.0)	-	0 (0.0)	0 (0.0)	-
Outpatient hospital visit	135 (0.7)	135 (1.3)	0 (0.0)	-	0 (0.0)	0 (0.0)	-
**ICU admission, n (%)**	6,405 (32.4)	3,029 (29.6)	3,376 (35.4)	< 0.0001	2,584 (43.2)	3,376 (35.4)	< 0.0001
Duration of ICU stay (days), mean ± SD [median]	3.7 ± 4.1 [2.0]	3.5 ± 4.1 [2.0]	3.9 ± 4.0 [3.0]	< 0.0001	3.6 ± 4.1 [2.0]	3.9 ± 4.0 [3.0]	< 0.0001
Time between admission and ICU transfer, mean ± SD [median]	0.8 ± 2.4 [0.0]	0.7 ± 2.7 [0.0]	0.8 ± 2.1 [0.0]	< 0.0001	0.6 ± 2.3 [0.0]	0.8 ± 2.1 [0.0]	< 0.0001
ICU transfer on the same day as admission, n (%)	4,774 (74.5)	2,322 (76.7)	2,452 (72.6)	0.0002	1,990 (77.0)	2,452 (72.6)	0.0001
**Mechanical ventilation, n (%)**	1,710 (8.6)	707 (6.9)	1,003 (10.5)	< 0.0001	667 (11.2)	1,003 (10.5)	0.2094

**Abbreviations:** ICU: intensive care unit; IED: invasive *Escherichia coli* disease; SD: standard deviation

Most patients required an inpatient stay at their index encounter (96.5%) with a mean duration of 6.9 days. A total of 8.6% required mechanical ventilation and 32.4% received medical services in an intensive care unit (ICU), of whom 74.5% were transferred to ICU on the day of admission. Among index encounters with an inpatient hospitalization, patients had a mean length of stay of 6.4 days in Group 1 IED, 7.2 days in Group 1 IED with sepsis, and 7.4 days in Group 2 IED, with 29.6%, 35.4%, and 43.2% of patients, respectively, transferred to ICU during their index encounter (Table 2).

### Treatments patterns and antibiotic resistance during index encounter

**Fig. 2:**
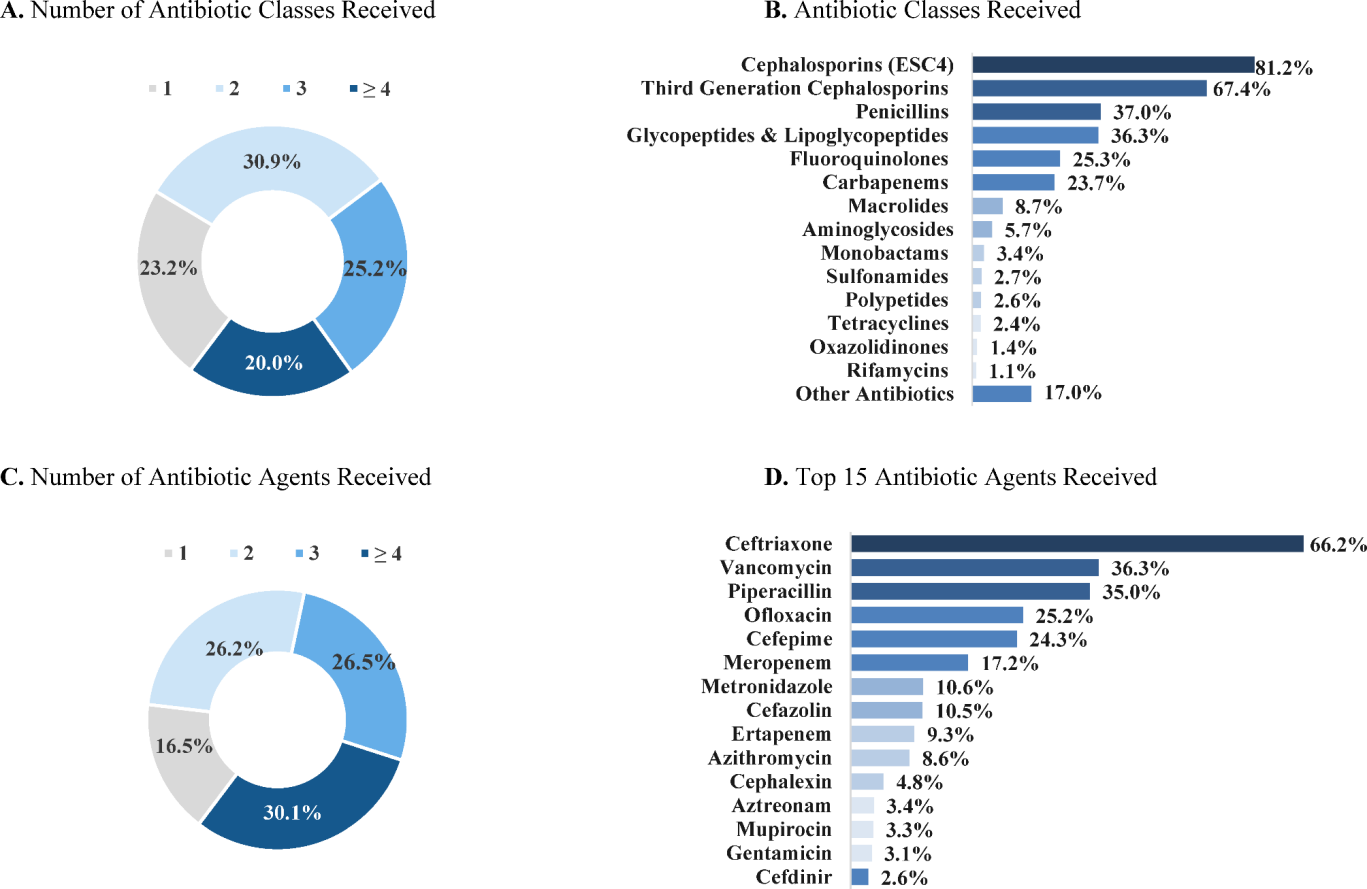
Antibiotic treatment during the index encounter at the class and agent level **Abbreviations**: IED: invasive Escherichia coli disease

Nearly all patients (99.3%) received antibiotic treatment and were typically treated with several antibiotic courses, with a mean of 2.9 different antibiotics. Notably, 30.1% of patients received ≥ 4 antibiotics. The most frequently observed antibiotics were ceftriaxone (66.2%), vancomycin (36.3%), and piperacillin (35.0%; Fig. 2). Of note, 87.9% of patients received ≥ 1 antibiotic prior to the confirmation of *E.coli* as the source of infection, with a mean of 1.57 different antibiotics per patient, which may explain why some patients received antibiotics not commonly used to treat *E. coli* infections (data not shown).

**Fig. 3:**
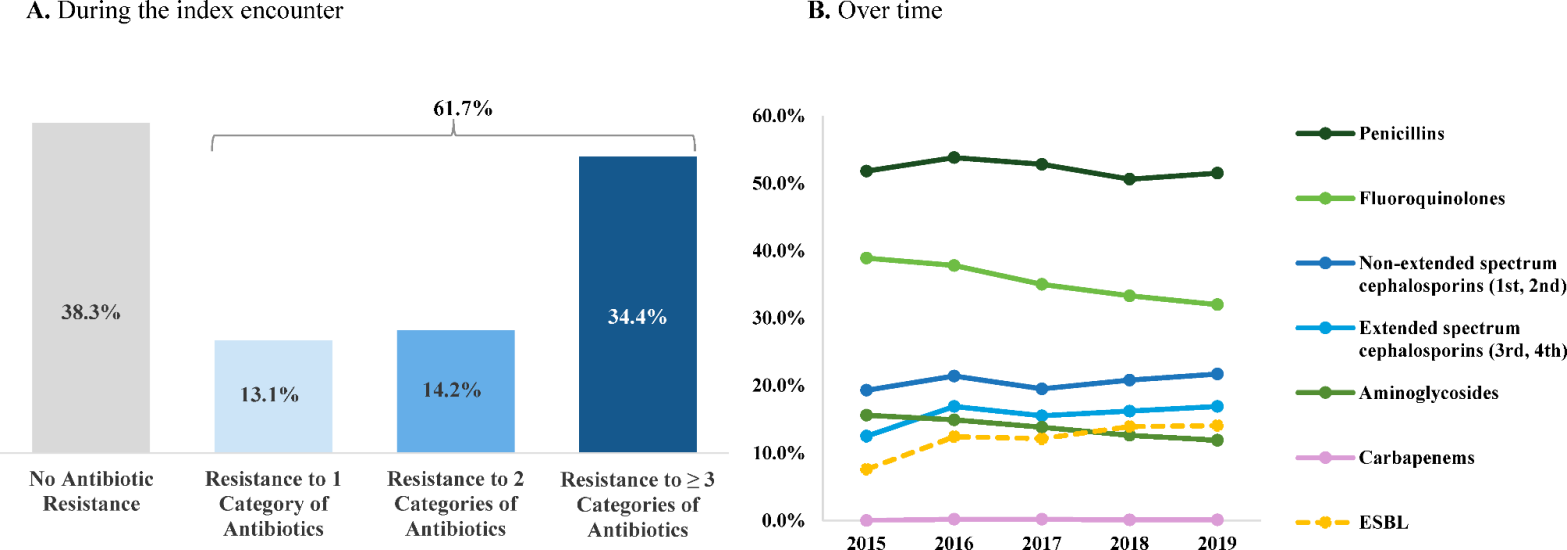
Patterns of antibiotic resistance during the index encounter and by agent over time **Abbreviations**: ICF: intermediate care facility; SNF: skilled nursing facility

During the index encounter, most patients had ≥ 1 antibiotic susceptibility test performed (98.0%). From nearly two-thirds of patients (61.7%), *E. coli* cultures displayed resistance to ≥ 1 antibiotic category, and 34.4% were resistant to ≥ 3 categories (i.e., MDR). Notably, rates for resistance of *E. coli* isolates to selected antibiotics were as follows: 51.7% to penicillins, 34.5% to fluoroquinolones, 20.5% to first and second generation non-extended spectrum cephalosporins, and 16.1% to third and fourth generation extended spectrum cephalosporins. Based on microbiology data, 13.3% of IED encounters were recorded as extended spectrum beta-lactamase (ESBL) positive. The proportion of *E. coli* isolates resistant to most of the antibiotic categories remained stable between 2015 and 2019, though a decreasing trend in the proportion of *E. coli* isolates resistant to fluoroquinolones (37.8–32.0%, p < 0.001) and aminoglycosides (15.6–11.9%, p = 0.002) was observed over time (Fig. 3).

### Point of origin, discharge status, and in-hospital death

**Fig. 4:**
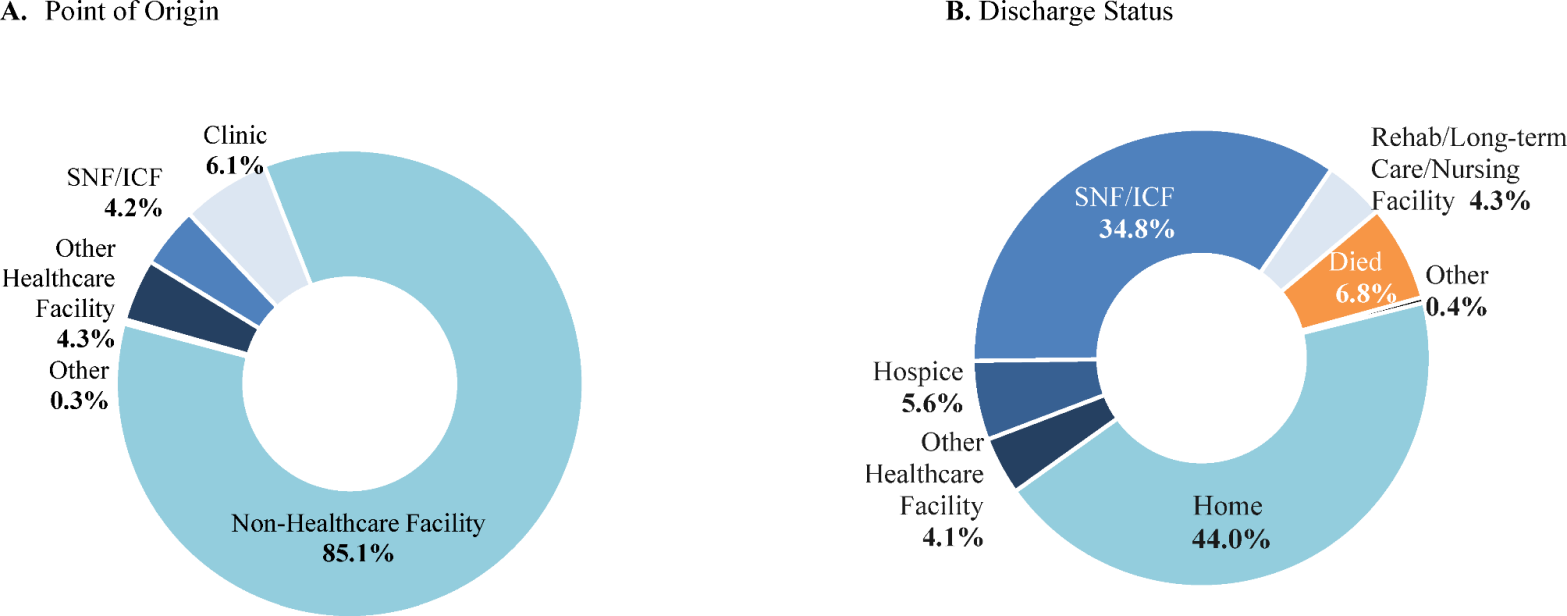
Point of origin and discharge status of the index encounter **Abbreviations:** ICF: intermediate care facility; SNF: skilled nursing facility

The most common point of origin was a non-healthcare facility (85.1%). In contrast, only 44% of patients were discharged home, while 34.8% were discharged to a skilled nursing facility (SNF) or an intermediate care facility (ICF). During the index encounter, 6.8% of patients died (Fig. 4), and the in-hospital fatality rate increased to 10.9% during the 12-month observation period; specifically, 3.6% of patients died within 2 days of the index date and 8.0% died within 1 month.

The in-hospital fatality rate during the index encounter was 6.6% in Group 1 IED, 9.6% in Group 1 IED with sepsis, and 7.0% in Group 2 IED. At 12 months post-index, 9.7% of patients in Group 1 IED died in the hospital relative to 13.1% in Group 1 IED with sepsis and 12.2% in Group 2 IED.

### Clinical outcomes post-IED

A total of 7,275 patients (36.8%) had ≥ 1 all-cause hospitalization during the 12-month observation period, of which 38.5% had a hospitalization related to invasive infectious disease based on primary diagnosis, and 21.9% and 34.4% of patients had an all-cause emergency room or outpatient hospital visit, respectively, during the same period. Of these, 477 patients (2.4%) had ≥ 1 IED recurrence, with a mean of 4.6 months between the index date and the first recurrence. During the observation period, 34.0%, 34.9%, 39.8% of patients in Group 1 IED, Group 1 IED with sepsis, and Group 2 IED, respectively, had ≥ 1 all-cause hospitalization (Table 3).

**Table 3:** IED during the 12-month observation period

	All patients	Type of IED at index date							
		Group 1	Group 2	P-value	Group 1 with sepsis	Group 2	P-value		
	N = 19,773	N = 10,235	N = 9,538		N = 5,978	N = 9,538			
**All-cause medical resource utilization (excluding index encounter**)									
Inpatient stays, mean ± SD [median]	0.7 ± 1.2 [0.0]	0.0 ± 1.1 [0.0]	0.7 ± 1.2 [0.0]	< 0.0001	0.6 ± 1.2 [0.0]	0.7 ± 1.2 [0.0]	< 0.0001		
0 stays, n (%)	12,498 (63.2)	6,752 (66.0)	5,746 (60.2)	< 0.0001	3,888 (65.0)	5,746 (60.2)	< 0.0001		
1 stay, n (%)	4,139 (20.9)	2,070 (20.2)	2,069 (21.7)	0.0113	1,216 (20.3)	2,069 (21.7)	0.0450		
≥ 2 stays, n (%)	3,136 (15.9)	1,413 (13.8)	1,723 (18.1)	< 0.0001	874 (14.6)	1,723 (18.1)	< 0.0001		
Number of inpatient days among patients who had ≥ 1 stay, mean ± SD [median]	10.6 ± 11.6 [7.0]	9.7 ± 10.8 [6.0]	11.4 ± 12.2 [7.0]	< 0.0001	10.2 ± 10.9 [6.0]	11.4 ± 12.2 [7.0]	< 0.0001		
Primary diagnosis recorded during an inpatient stay, n (%)									
**Invasive infectious disease-related**	2,799 (38.5)	1,458 (41.9)	1,341 (35.4)	< 0.0001	880 (42.1)	1,341 (35.4)	< 0.0001		
IED recurrence	465 (6.4)	221 (6.3)	244 (6.4)	0.8761	135 (6.5)	244 (6.4)	0.9705		
Other infectious invasive disease (i.e., not IED)	2,456 (33.8)	1,294 (37.2)	1,162 (30.6)	< 0.0001	783 (37.5)	1,162 (30.6)	< 0.0001		
Other infectious disease	119 (1.6)	63 (1.8)	56 (1.5)	0.2648	41 (2.0)	56 (1.5)	0.1622		
Other disease-related									
Neoplasms	269 (3.7)	160 (4.6)	109 (2.9)	0.0001	91 (4.4)	109 (2.9)	0.0027		
Diseases of the blood and blood-forming organs and certain disorders involving the immune mechanism	169 (2.3)	79 (2.3)	90 (2.4)	0.7659	59 (2.8)	90 (2.4)	0.2936		
Endocrine, nutritional, and metabolic diseases	412 (5.7)	166 (4.8)	246 (6.5)	0.0015	99 (4.7)	246 (6.5)	0.0062		
Mental, behavioral, and neurodevelopmental disorders	88 (1.2)	21 (0.6)	67 (1.8)	< 0.0001	15 (0.7)	67 (1.8)	0.0010		
Diseases of the nervous system	300 (4.1)	120 (3.4)	180 (4.7)	0.0053	72 (3.4)	180 (4.7)	0.0183		
Diseases of the eye and adnexa	3 (0.0)	2 (0.1)	1 (0.0)	0.6097	1 (0.0)	1 (0.0)	-		
Diseases of the ear and mastoid process	3 (0.0)	2 (0.1)	1 (0.0)	0.6097	2 (0.1)	1 (0.0)	0.2890		
Diseases of the circulatory system	1,620 (22.3)	678 (19.5)	942 (24.8)	< 0.0001	413 (19.8)	942 (24.8)	< 0.0001		
Diseases of the respiratory system	1,049 (14.4)	448 (12.9)	601 (15.8)	0.0003	265 (12.7)	601 (15.8)	0.0010		
Diseases of the digestive system	1,035 (14.2)	526 (15.1)	509 (13.4)	0.0406	334 (16.0)	509 (13.4)	0.0074		
Diseases of the skin and subcutaneous tissue	204 (2.8)	88 (2.5)	116 (3.1)	0.1693	54 (2.6)	116 (3.1)	0.2976		
Diseases of the musculoskeletal system and connective tissue	245 (3.4)	131 (3.8)	114 (3.0)	0.0746	74 (3.5)	114 (3.0)	0.2648		
Diseases of the genitourinary system	1,535 (21.1)	680 (19.5)	855 (22.5)	0.0016	392 (18.8)	855 (22.5)	0.0007		
Emergency room visits, mean ± SD [median]	0.4 ± 1.1 [0.0]	0.4 ± 1.0 [0.0]	0.4 ± 1.2 [0.0]	0.0052	0.4 ± 0.9 [0.0]	0.4 ± 1.2 [0.0]	0.7793		
0 visits, n (%)	15,437 (78.1)	7,908 (77.3)	7,529 (78.9)	0.0045	4,705 (78.7)	7,529 (78.9)	0.7310		
1 visit, n (%)	2,673 (13.5)	1,433 (14.0)	1,240 (13.0)	0.0398	788 (13.2)	1,240 (13.0)	0.7447		
≥ 2 visits, n (%)	1,663 (8.4)	894 (8.7)	769 (8.1)	0.0888	485 (8.1)	769 (8.1)	0.9104		
Outpatient hospital visits, mean ± SD [median]	1.6 ± 5.1 [0.0]	1.9 ± 5.8 [0.0]	1.4 ± 4.1 [0.0]	< 0.0001	1.8 ± 4.6 [0.0]	1.4 ± 4.1 [0.0]	< 0.0001		
0 visits, n (%)	12,974 (65.6)	6,237 (60.9)	6,737 (70.6)	< 0.0001	3,708 (62.0)	6,737 (70.6)	< 0.0001		
1 visit, n (%)	2,292 (11.6)	1,311 (12.8)	981 (10.3)	< 0.0001	736 (12.3)	981 (10.3)	< 0.0001		
≥ 2 visits, n (%)	4,507 (22.8)	2,687 (26.3)	1,820 (19.1)	< 0.0001	1,534 (25.7)	1,820 (19.1)	< 0.0001		
**IED recurrence, n (%)**	477 (2.4)	229 (2.2)	248 (2.6)	0.0967	140 (2.3)	248 (2.6)	0.3161		
Duration from index date to first recurrence (months), mean ± SD [median]	4.6 ± 3.3 [3.6]	4.5 ± 3.3 [3.4]	4.7 ± 3.4 [3.7]	0.8018	4. 6 ± 3.3 [3.5]	4.7 ± 3.4 [3.7]	0.9756		

**Abbreviations:** IED: invasive *Escherichia coli* disease; SD: standard deviation

### Further stratified analyses

Patient characteristics varied by age; compared to patients 60–75 years old, those in the ≥ 75 years old subgroup had a more severe comorbidity profile based on a Charlson Comorbidity Index (CCI) score ≥ 3 (45.3% vs. 38.4%, p < 0.001), and were less likely to be discharged to their home (35.2% vs. 56.7%, p < 0.001) and more likely to be discharged to a SNF or ICF (42.0% vs. 24.2%, p < 0.001). Further, a higher proportion of patients ≥ 75 years old died during the index encounter (7.3% vs. 6.1%, p < 0.001) and at 12 months post-index (11.8% vs. 9.6%; p < 0.001).

Patients with hospital-onset IED tended to have a more severe comorbidity profile compared to those with community-onset IED (CCI score ≥ 3: 56.4% vs. 41.6%, p < 0.001). Compared to patients with community-onset IED, those with hospital-onset IED were more likely to receive care in a teaching hospital (53.6% vs. 39.2%, p < 0.001). A higher proportion of patients with hospital-onset IED died during the index encounter (13.3% vs. 6.4%, p < 0.001) and at 12 months post-index (19.6% vs. 10.4%, p < 0.001) compared to those with community-onset IED. The proportion of encounters that required ICU transfer was greater among hospitalonset IED (53.0% vs. 31.2%, p < 0.001), with a longer mean duration (6.7 days vs. 3.4 days, p < 0.001).

Patients with MDR isolates tended to have a more severe comorbidity profile compared to those with non-MDR isolates (CCI score ≥ 3: 46.7% vs. 40.3%, p < 0.001). Encounters with MDR isolates were more likely to be associated with hospital-onset IED (6.5% vs. 5.3%, p < 0.001), occur in hospitals of ≥ 500 beds (33.2% vs. 29.1%, p < 0.001), and originate from a SNF/ICF (5.2% vs. 3.6%, p < 0.001) compared to non-MDR isolates. For their index encounter, a higher proportion of patients with MDR than non-MDR isolates received ≥ 4 agents (33.9% vs. 28.1%, p < 0.001). During the 12-month observation period, the proportion of patients who had ≥ 1 IED recurrence was higher among those with MDR isolates (4.1% vs. 1.5%, p < 0.001). The proportion of patients who had ≥ 1 hospitalization during this period was also higher among those with MDR isolates (40.8% vs. 34.8%, p < 0.001). The rate of inhospital death was not statistically different between patients with MDR and non-MDR isolates (11.5% vs. 10.6%, p = 0.070).

## Discussion

*E. coli* is the most commonly reported pathogen leading to hospitalizations for sepsis in older adults in the U.S. [5, 7]. Considering the epidemiological data showing an increased incidence of *E. coli* infection worldwide [5, 20, 21], it is important to characterize the course of IED in U.S. hospitals. The results of the current study highlight the substantial burden associated with IED in the U.S. in terms of hospitalizations, ICU admissions, and in-hospital fatality rates. Almost all index encounters led to hospitalization and nearly 1 in 3 patients were admitted to ICU. In addition to the acute burden observed at the index encounter, patients continued to experience poor outcomes up to one year post-encounter. More than 1 in 10 patients died in a hospital within a year of their first IED encounter, with the majority of deaths occurring within the first month post-IED. Further, while most IED originated in a non-healthcare facility–and only 4.2% through SNF/ICF–approximately one-third of patients were discharged to an SNF/ICF after their index encounter, underscoring the long-term consequences among those who survive IED. Moreover, older patients, who had a more severe comorbidity profile, experienced a higher burden of IED and were less likely to be discharged to their home and more likely to be discharged to a SNF/ICF. Together, these results highlight the importance of maintaining continuity of care after the index encounter.

While published data on the clinical burden of IED is limited, our findings are consistent with a recent publication using similar administrative data from the PHD database by Begier et al. [14]. However, Begier et al. described a more limited range of clinical outcomes and focused on an IED subtype with microbiological confirmation from blood or other normally sterile body sites. In many instances, clinical sepsis cases lack confirmation from a positive blood culture. Sensitivity and specificity of blood culture depends on blood volume drawn, timing, prior treatment with antibiotics, and the presence of viable organisms [22]. Fay et al. 2020 reported that a specific pathogen was identified in only 56.9% of sepsis cases, leaving almost half of sepsis cases with an unidentified infection source [6]. A recent publication (Rhee et al., 2020) reporting on community-onset sepsis found that urine was the most common source of positive culture, allowing for pathogen identification in 52% of patients [5]. Therefore, microbiological confirmation from sources other than blood culture (i.e., urine culture) are deemed important to appropriately capture the full burden of IED, especially for community-onset sepsis. As such, the present study incorporated a two-part definition of IED, whereby encounters were considered as an IED event if they included a positive *E. coli* culture in urine with UTI and signs of sepsis (i.e., Group 2 IED) [16], in addition to IED identified from a positive *E. coli* culture in blood or other normally sterile body sites (i.e., Group 1 IED). Findings from this study suggest that patients who presented with sepsis of likely urinary tract origin (Group 2; i.e., non-bacteremic urosepsis), though lacking microbiological confirmation from normally sterile body sites, can incur a substantial clinical burden comparable to those who present with bacteremic disease and microbiological confirmation from normally sterile body sites.

This study also provides a comparison of the burden of IED between those with community- vs. hospital-onset. Consistent with previous literature, most patients acquired IED in a community setting [11, 14]. Though this resulted in a substantial burden, patients with hospital-onset IED incurred a significant burden, including a higher rate of ICU admissions and in-hospital fatality compared to community-onset IED, which confirms prior research [23].

Antibiotic treatment patterns also suggest that IED can be complex to manage and involve a broad range of antibiotics being received within a short timeframe. For example, more than 1 in 4 patients were treated with ≥ 4 agents during their index encounter. A high rate of antibiotic resistance was observed in our study sample, with MDR isolates being observed in more than 1 in 3 index encounters. Though the exact patterns of antibiotic resistance in *E. coli* isolates reported in recent literature vary depending on the study population (i.e., target age, country), disease definition, or study design, resistance to penicillins and fluoroquinolones has been consistently high [23–25]. In the current study, more than half of the patient population had ExPEC that was resistant to penicillins consistently over time. A high level of resistance was also observed for fluoroquinolones, though this appears to be trending downwards over time (38% in 2015 to 32% in 2019). Furthermore, MDR isolates were associated with an increased number of antibiotic agents received and higher incidence of IED recurrence, which supports prior evidence of the association between inadequate treatment and resistant pathogens [6, 26, 27]. Antibiotic resistance may lead to treatment failure, increased rates of hospitalization, morbidity, mortality, and associated costs [2, 28–32], and can drive the evolution of novel pathogenic clones, such as ST131 [33].

The use of the PHD database, which encompasses detailed admission-level data of inpatient services for patients admitted to over 1,000 U.S. hospitals, is an important strength of this study as it provides a large representative sample from all U.S. regions. Importantly, the database includes microbiology laboratory data, which is not available in most other administrative claims databases, with information on specimen source, tests performed, and results for these tests that allow for the identification of IED encounters. The study relied on the CDC’s clinical surveillance definition for sepsis, which has been previously validated, and demonstrates its value for research purposes.

This retrospective study is subject to inherent limitations. IED encounters were identified based on microbiological data from laboratory records and diagnosis and procedure codes in claims data; therefore, some patients may have been misidentified as having IED due to any limitations in the various data sources (e.g., coding errors, etc.). Furthermore, the definition of IED used in this study included sepsis, for which a range of definitions exist in the literature; these may affect epidemiological estimates of sepsis by as much as three-fold [34, 35]. Comparisons with other studies that use different definitions of IED and sepsis are therefore inherently limited. Further, no information on prescription fills was available in the database, thus antibiotic use was identified based on the medications received in the hospital setting only. It should also be noted that, specimens are not systematically tested for resistance to all possible antibiotics in real-world clinical practice; therefore, MDR incidence may have been underestimated. Additionally, information in the PHD database is limited to IED encounters occurring in a hospital setting such that medical resource utilization for a given patient is only captured for encounters at a given hospital. Similarly, since death was identified based on discharge status, deaths occurring outside of the hospital also were not captured. Finally, this study is descriptive in nature, such that no causal inference can be made.

## Conclusions

This study described the course of IED in U.S. hospitals among a large representative sample of older adults. The findings suggest that IED is associated with an acute burden during the initial hospital encounter and may lead to poor outcomes even after the encounter is resolved. This burden is particularly high in the presence of antibiotic resistance, which is an important consideration for an increasing aging population.

### Electronic supplementary material

Below is the link to the electronic supplementary material.

Supplementary Material 1

## Data Availability

The data that support the findings of this study are available from PREMIER, but restrictions apply to the availability of these data, which were used under license for the current study, and so are not publicly available. Data are, however, available from the corresponding author upon reasonable request and with permission of PREMIER.

## Declarations

### Previous presentations

A portion of these results were presented at the IDWeek conference held in Washington, DC, USA, October 19–23, 2022.

## Abbreviations

CDC: Centers for Disease Control
CCI: Charlson Comorbidity Index
*E. coli*: *Escherichia coli*
ExPEC: Extraintestinal pathogenic *E. coli*
ICF: Intermediate care facility
InPEC: Intestinal pathogenic *E. coli*
IED: Invasive *Escherichia coli* disease
MDR: Multi-drug resistance
SNF: Skilled nursing facility
SIRS: Systemic inflammatory response syndrome
U.S.: United States
