# Supplementary material for: Clinical burden of invasive Escherichia coli disease among older adult patients treated in hospitals in the United States
Source: BMC Infect Dis. 2023 Aug 22;23:550. doi: 10.1186/s12879-023-08479-3 (PMC10464165; doi:10.1186/s12879-023-08479-3)
Supplement: Supplementary file 1 — Supplementary Material 1 [file 12879_2023_8479_MOESM1_ESM.docx]

**Supplementary materials**

*Figure S1. Study design*

**End of
Observation Period**

**Index Date**

**First IED encounter**

Patient was 60 years or older

≥ 6 months

≥ 12 months

Hospital continuously contributed microbiology data

**12-Month Observation Period:**Assessment of clinical course of IED

**Abbreviation:** IED: invasive extraintestinal pathogenic *E. coli* (ExPEC) disease

*Figure S2. Sample selection*

**Abbreviations:** IED: invasive extraintestinal pathogenic *E. coli* (ExPEC) disease; SIRS: systemic inflammatory response syndrome

**Notes**:

a. IED encounters were identified based on either (Group 1 IED) a positive *E. coli* culture in either blood or in any other normally sterile body site and hospitalization with signs of sepsis or SIRS, and no positive culture for other bacteria or fungal pathogens, or (Group 2 IED) a positive *E. coli* culture in urine with signs of sepsis and a diagnosis for UTI, with no positive culture for *E. coli* in blood or normally sterile body sites and no positive culture for other bacteria or fungal pathogens.

b. The index date was defined as the date of the first documented IED encounter.

*Table S1. IED infection types*

| **Variables** | **Definitions** |
| --- | --- |
| **Complicated pneumonia** | A. Having a positive or presumptively positive *E. coli* culture from bronchial/BAL lavage or from sputum/respiratory/pneumonia AND having positive or presumptively positive *E. coli* culture from blood during the same hospital visit |
| **Wound/surgical site infection** | A. Having positive or presumptively positive *E. coli* culture from a deep wound;  **OR**  B. Having positive or presumptively positive *E. coli* culture from a normally sterile site culture AND having ICD-10-CM code of T81.4 (infection following a procedure) during the same hospital visit;  **OR**  C. Having positive or presumptively positive *E. coli* culture from a wound AND having positive or presumptively positive *E. coli* culture from a normally sterile site during the same hospital visit |
| **Meningitis** | A. Having positive or presumptively positive *E. coli* culture from CSF;  **OR**  B. Having positive or presumptively positive *E. coli* culture from a normally sterile site AND having ICD-10-CM codes of G01 or G00.9 (meningitis in bacterial diseases classified elsewhere or bacterial meningitis, unspecified) during the same hospital visit |
| **Neutropenic fever** | Having IED and having ICD-10-CM code of D70.9 (neutropenia, unspecified) during the same hospital visit |
| **Osteomyelitis** | A. Having positive or presumptively positive *E. coli* culture from bone;  **OR**  B. Having positive or presumptively positive *E. coli* culture from a normally sterile site culture AND having ICD-10-CM code of M86 (osteomyelitis) during the same hospital visit |
| **Cholangitis** | A. Having positive or presumptively positive *E. coli* culture from gallbladder, bile, biliary tract;  **OR**  B. Having positive or presumptively positive *E. coli* culture from a normally sterile site AND having ICD-10-CM code of K83.0 (cholangitis) during the same hospital visit |
| **Peritonitis** | A. Having a positive or presumptively positive *E. coli* culture from peritoneal fluid;  **OR**  B. Having positive or presumptively positive *E. coli* culture from a normally sterile site culture AND having ICD-10-CM code of K65 (peritonitis) during the same hospital visit |
| **Other intra-abdominal infection** | Having a positive or presumptively positive *E. coli* culture from peritoneal fluid AND do not meet the definition of any other syndrome in this table (except other IED) |
| **Prostate biopsy-related infection** | Having a positive or presumptively positive *E. coli* culture from a normally sterile site AND having ICD-10-PCS code of 0V9xxx or CPT codes of 55706 (biopsies, prostate, needle, transperineal), 55700 (prostate needle biopsy), during the same hospital visit or with hospital admission date within 30 days of specimen collection date |
| **Urosepsis with bacteremia** | Having a positive or presumptively positive *E. coli* culture from blood AND having ICD code for pyelonephritis (ICD-10-CM N10 [Infections of kidney/Pyelonephritis] or ICD-10-CM N39.0 [UTI, site not specified]) during the same hospital visit or with specimen collection date within 7 calendar days of hospital admission date with the aforementioned codes |
| **Urosepsis without bacteremia** | Having a positive or presumptively positive *E. coli* culture from urine AND having sepsis, as per the definition in Rhee et al. (2017) [14] AND having ICD code for UTI AND no positive culture for other bacteria or fungal pathogens |
| **Other blood stream infection** | Having positive or presumptively positive *E. coli* culture from blood AND do not meet the definition of any other syndrome in this table (except other IED) |
| **Other IED** | Having IED AND do not meet any of the above definitions |

**Abbreviations:** CPT: Current Procedural Terminology; CSF: cerebrospinal fluid; ICD-10-CM: International Classification of Disease, Tenth Revision, Clinical Modification; ICD-10-PCS: International Classification of Disease, Tenth Revision, Procedure Coding System; IED: invasive ExPEC disease

*Table S2. Antibiotic classes and agents*

| **Class** | **Category^a^** | **Agent** |
| --- | --- | --- |
| Aminoglycoside | Aminoglycoside | Amikacin |
|  |  | Gentamicin |
|  |  | Tobramycin |
|  |  | Netilmicin |
|  |  | Kanamycin^b^ |
| β-lactams | Non-extended spectrum cephalosporin: first and second generation | Cefaclor^b^ |
|  |  | Cefadroxil^b^ |
|  |  | Cefalexin^b^ |
|  |  | Cefazolin |
|  |  | Cefuroxime |
|  |  | Cephalothin^b^ |
|  |  | Cefamandole^b^ |
|  |  | Cefmetazole^b^ |
|  |  | cefonicid^b^ |
|  | Extended spectrum cephalosporin: third and fourth generation | Cefixime^b^ |
|  |  | Ceftibuten^b^ |
|  |  | Cefotaxime |
|  |  | Cefpodoxime^b^ |
|  |  | Ceftriaxone |
|  |  | Ceftazidime |
|  |  | Cefepime |
|  |  | Ceftazidime/avibactam |
|  |  | Ceftaroline/avibactam |
|  |  | Ceftolazane/tazobactam |
|  | Anti-MRSA cephalosporin | Ceftaroline |
|  |  | Ceftobiprole^b^ |
|  | Cephamycin | Cefoxitin |
|  |  | Cefotetan |
|  | Penicillin | Amoxicillin^b^ |
|  |  | Ampicillin |
|  |  | Mecillinam^b^ |
|  |  | Piperacillin^b^ |
|  |  | Ticarcillin^b^ |
|  |  | Carbenicillin^b^ |
|  |  | Mezlocillin^b^ |
|  | Penicillin + β-lactamase inhibitor | Amoxicillin/Clavulanate |
|  |  | Ampicillin/Sulbactam |
|  | Penicillin + β-lactamase inhibitor: antipseudomonal | Ticarcillin/Clavulanate |
|  |  | Piperacillin/Tazobactam |
|  | Monobactam | Aztreonam |
|  | Carbapenem | Ertapenem |
|  |  | Imipenem |
|  |  | Meropenem |
|  |  | Doripenem |
| Fluoroquinolone | Fluoroquinolone | Ciprofloxacin |
|  |  | Levofloxacin^b^ |
|  |  | Moxifloxacin^b^ |
|  |  | Ofloxacin^b^ |
|  |  | Perfloxacin^b^ |
|  |  | Norfloxacin^b^ |
| Folate pathway inhibitor | Folate pathway inhibitor | Trimethoprim^b^ |
|  |  | Sulfamethoxazole^b^ |
|  |  | Trimethoprim-sulfamethoxazole |
| Phenicol | Phenicol | Chloramphenicol |
| Phosphonic acid | Phosphonic acid | Fosfomycin |
| Polymyxin | Polymyxin | Colistin |
| Glycylcycline | Glycylcycline | Tigecycline |
| Tetracycline | Tetracycline | Eravacycline^b^ |
|  |  | Doxycycline |
|  |  | Minocycline |
|  |  | Tetracycline |

**Notes:**

a. Antibiotic categories considered for multi-drug resistance are based on a joint initiative by the U.S. and the European Centre for Disease Prevention and Control, as reported in Magiorakos et al. (2012) [17].

b. Denotes agents that were included in this study but were not originally included in Magiorakos *et al.* (2012) [17].
